# Phyllospheric microbial community structure and carbon source metabolism function in tobacco wildfire disease

**DOI:** 10.3389/fcimb.2024.1458253

**Published:** 2024-11-01

**Authors:** Xia Xu, Liang Zhao, Yanfei Chen, Hancheng Wang, Liuti Cai, Yanyan Wang, Nalin N. Wijayawardene, Weihua Pan, Feng Wang, Yingqian Kang

**Affiliations:** ^1^ Key Laboratory of Environmental Pollution Monitoring and Disease Control, Ministry of Education of Guizhou & Institute of Health Research & Key Laboratory of Medical Microbiology and Parasitology, School of Basic Medical Sciences, Guizhou Medical University, Guiyang, China; ^2^ Guizhou Provincial Academician Workstation of Microbiology and Health, Guizhou Academy of Tobacco Science, Guiyang, China; ^3^ Center for Yunnan Plateau Biological Resources Protection and Utilization, College of Biological Resource and Food Engineering, Qujing Normal University, Qujing, Yunnan, China; ^4^ Department of Dermatology, Changzheng Hospital, Shanghai, China

**Keywords:** tobacco wildfire disease, microbial community, high-throughput sequencing, biolog-eco, disease severity

## Abstract

The phyllospheric microbial composition of tobacco plants is influenced by multiple factors. Disease severity level is one of the main influencing factors. This study was designed to understand the microbial community in tobacco wildfire disease with different disease severity levels. Tobacco leaves at disease severity level of 1, 5, 7, and 9 (L1, L5, L7, and L9) were collected; both healthy and diseased leaf tissues for each level were collected. The community structure and diversity in tobacco leaves with different disease severity levels were compared using high-throughput technique and Biolog Eco. The results showed that in all healthy and diseased tobacco leaves, the most dominant bacterial phylum was Proteobacteria with a high prevalence of genus *Pseudomonas*; the relative abundance of *Pseudomonas* was most found at B9 diseased samples. Ascomycota represents the most prominent fungal phylum, with *Blastobotrys* as the predominant genus. In bacterial communities, the Alpha diversity of healthy samples was higher than that of diseased samples. In fungal community, the difference in Alpha diversity between healthy and diseased was not significant. LEfSe analysis showed that the most enriched bacterial biomarker was unclassified_Gammaproteobacteria in diseased samples; unclassified_Alcaligenaceae were the most enrich bacterial biomarker in healthy samples. FUNGuild analysis showed that saprotroph was the dominated mode in health and lower diseased samples, The abundance of pathotroph–saprotroph and pathotroph–saprotroph–symbiotroph increases at high disease levels. PICRUSt analysis showed that the predominant pathway was metabolism function, and most bacterial gene sequences seem to be independent of the disease severity level. The Biolog Eco results showed that the utilization rates of carbon sources decrease with increasing disease severity level. The current study revealed the microbial community’s characteristic of tobacco wildfire disease with different disease severity levels, providing scientific references for the control of tobacco wildfire disease.

## 1 Introduction

Tobacco (*Nicotiana tabacum* L.) is an extensively cultivated economic crop in China, whose growth and production are affected by a myriad of biotic and abiotic factors (Ma et al., 2023). Tobacco wildfire disease is caused by *Pseudomonas syringae* pv. *tabaci*, which results in heavy economical losses (Tumewu et al., 2022). The typical symptoms of tobacco wildfire disease include small brown or black lesions bordered by chlorotic halos on tobacco leaves (Sun et al., 2021). The characteristic chlorotic halos are produced by a toxin, tabtoxin (Ichinose et al., 2013).

The phyllosphere is an important habitat for many microorganisms, including commensal, beneficial, and pathogenic microbes affecting plant health and productivity (Lindow and Brandl, 2003; Xin et al., 2018; Zhu et al., 2022). It is notable to study the changes in microbial communities (natural inhabitants) when the host plant is stressed under an infection (Perreault and Laforest-Lapointe, 2022). The development of PCR and sequencing technologies (including high-throughput sequencing) has facilitated the exploration of microbial diversity and structure of microbial communities (Ahmed et al., 2022b; Fitzpatrick et al., 2020; Gong and Xin, 2021).

Tobacco bacterial wilt is caused by *Ralstonia solanacearum* and is reported to reduce the leaf fungal community abundance and diversity (Ahmed et al., 2022a), whereas a similar response was observed by Si et al. (2023) during the investigation of tobacco wildfire disease caused by *P. syringae* pv. *tabaci*. Chen et al. (2020a) noticed a much higher abundance of *Rhizopus oryzae* in cured tobacco leaves infected by tobacco pole rot than that of healthy leaves. The phyllospheric microbial community and composition are closely related to plant health (Zhang et al., 2019).

Disease severity level also affects the leaf microbiome, influencing the dynamics of phyllosphere microbial communities. Huang et al. (2021) studied the influence of the disease severity level on the community structure and diversity of the tobacco leaf spot disease caused by *Didymella segeticola*, identifying that the community diversity of phyllosphere fungi decreased with the increase in the disease severity level. Sun et al. (2022) studied the microbial community of tobacco leaves affected by target spot at different disease severity levels. The results indicate that fungal diversity and relative abundance increased with disease severity level, while bacterial diversity indices decreased. Luo et al. (2019) studied the microbial community structure of symptomatic cucumber leaves affected by angular leaf spot at different disease severity levels. The investigation showed that the pathogen caused an increase in the microbial community richness when disease pressure was higher. Biolog Eco can reflect the main types of carbon source utilization and the strength of metabolic functions of all microbial communities (Rutgers et al., 2016). This technique has been used to identify the carbon-source-associated metabolic pathways of phyllosphere microbial communities in both healthy and diseased tobacco plants (Feng et al., 2023; Shen et al., 2023). However, the effect of disease severity level on the structure and diversity of tobacco wildfire disease phyllosphere microbial community is still poor understood.

Tobacco wildfire disease can lead to serious damage. It is important to understand the microbial community changes in tobacco leaves. Therefore, the goals of this study were to identify the structure and diversity of phyllosphere bacterial and fungal communities and the metabolism of carbon sources of tobacco microorganisms in tobacco leaves with different disease severity levels. These results will provide theoretical reference for the prevention and control of wildfire disease in tobacco.

## 2 Materials and methods

### 2.1 Sampling sites and sampling strategy

**Figure 1:**
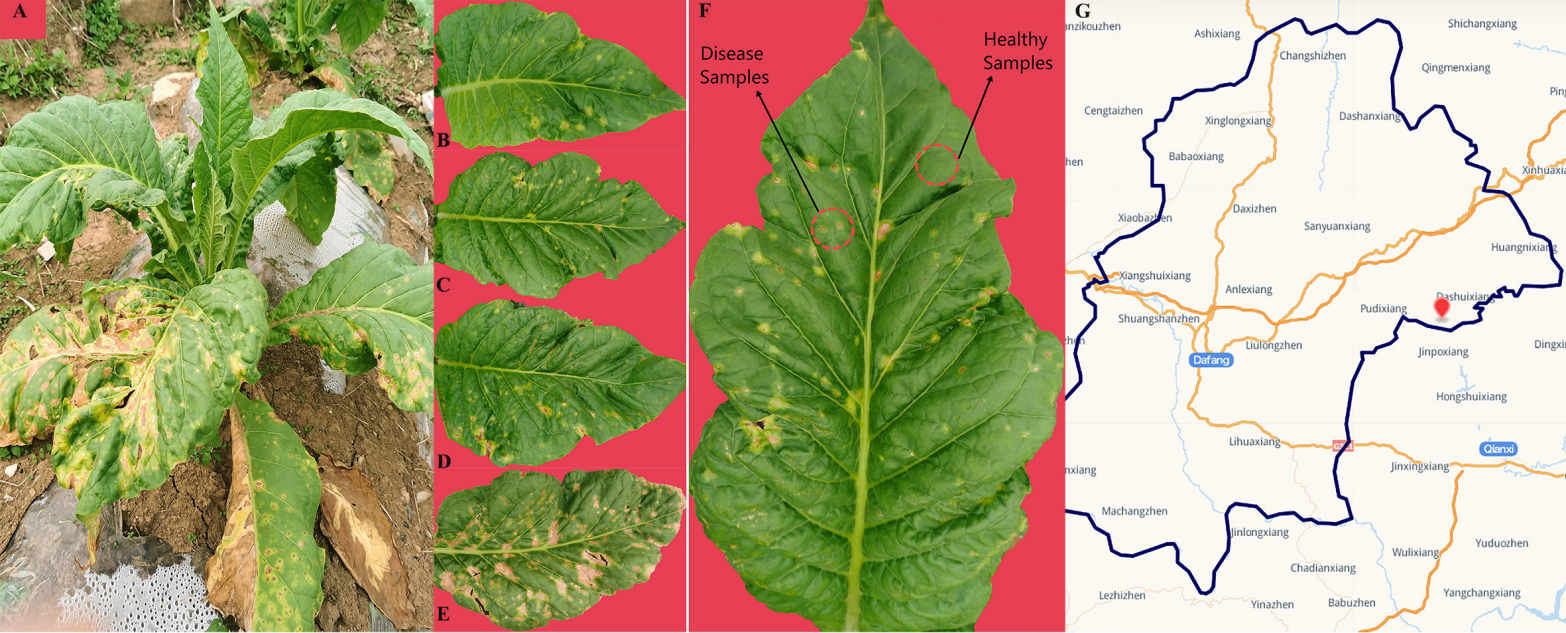
Whole plant **(A)** and leaves displaying disease severity at **(B)** L1, **(C)** L5, **(D)** L7, and **(E)** L9, respectively. **(F)** sampling parts display of diseased and healthy samples on tobacco leaves. **(G)** map of sampling site.

In Jun 2022, one tobacco field (cultivar Yunyan 87) with symptoms of tobacco wildfire disease in Dafang County (27°12′15″ N, 105°57′18″ E), Bijie City, Guizhou Province of China, was selected as the sampling site. The tobacco leaves of different disease severity levels (L1, L5, L7, and L9) were randomly selected based on the Chinese National Standard (GB/T 23222-2008). For L1, L5, L7, and L9 groups, diseased lesion area ranged from 0% to 1%, 6% to 10%, 11% to 20%, and 11% to 100% of a leaf, respectively (**Figures 1A–E**). There were three biological replicates from each group. A total of 24 samples were collected. The four groups of leaves were separated into two parts, with and without visible leaf spots (**Figure 1F**), and labeled as diseased and healthy samples, respectively, as presented in **Table 1**. After sampling, the samples were immediately transported to the laboratory and stored at −80°C until further use.

**Table 1:** Sample information for both diseased and healthy tobacco leaves with infected tobacco wildfire disease at different disease severity.

Disease severity	Proportion of lesion area every leaf	Diseased groups (sample)	Healthy groups (sample)
L1	≤1%	B1 (B11, B12, B13)	J1 (J11, J12, J13)
L5	6%–10%	B5 (B51, B52, B53)	J5 (J51, J52, J53)
L7	11%–20%	B7 (B71, B72, B73)	J7 (J71, J72, J73)
L9	≥21%	B9 (B91, B92, B93)	J9 (J91, J92, J93)

### 2.2 DNA extraction, PCR amplification, and high-throughput sequencing

Total DNA was extracted from the leaf samples using the PF Mag-Bind Stool DNA Kit (Omega Bio-Tek, GA, USA) according to the manufacturer’s instructions. The quality and concentration of DNA were determined by agarose gel electrophoresis. The hypervariable regions V5–V7 of the bacterial 16S rRNA gene were amplified with primer pairs 799F (5′-AACMGGATTAGATACCCKG-3′) and 1193R (5′-ACGTCATCCCCACCTTCC-3′) (Bulgarelli et al., 2015). Furthermore, the primer pair ITS1F (5′-CTTGGTCATTTAGAGGAAGTAA-3′) and ITS2R (5′-GCTGCGTTCTTCATCGATGC-3′) (Adams et al., 2013) were used to amplify the fungal ITS1 region. PCR products were checked by 2% agarose gel electrophoresis. The amplification products were amplified using an Illumina MiSeq platform and constructed the library.

### 2.3 Sequencing data processing

Qualitative filtering and merging of raw sequencing reads were performed using Flash (version 1.2.11) (Magoč and Salzberg, 2011). All sequences were clustered into operational taxonomic units (OTUs) by Uparse (version 11) (Edgar, 2013) with a 97% similarity cutoff. Subsequently, the OTUs were annotated for bacterial and fungal based on the Silva 16S rRNA (v138) and Unite (version 8.0) database, respectively, setting the confidence threshold to 70% (Huang et al., 2021; Sun et al., 2022). The Alpha and Beta diversities were performed by Mothur (version 1.30.2) and Qiime (2020.2.0), and the R statistics package (version 3.3.1) was utilized to draw the principal coordinate analysis (PCoA) diagram. The linear discriminant analysis (LDA) effect size (LEfSe) (Segata et al., 2011) was performed to detect the potential biomarker, using a linear discriminant analysis threshold of 4.0. The PERMANOVA test was used to calculate significant differences. PICRUSt and FUNGuild (Douglas et al., 2020) the databases were used to analyze enzyme functional and fungal trophic mode. Bacterial metabolic functions were analyzed with the bioinformatics software package PICRUSt (Barberán et al., 2012). The correlation of phyllosphere microorganisms was demonstrated using Spearman’ s rank analysis based on the absolute value of a correlation coefficient >0.6 and a *p*-value <0.05 (Nguyen et al., 2016).

### 2.4 Biolog inoculation and analysis

Each of the 1-g samples from different disease severity levels was mixed with 50 ml of 0.85% NaCl and shaken (180 rpm) for 2 h at 28°C. The mixture was standing for 30 min, and 100 µL of dilution was added using an electronic pipette and incubated at 28°C in a Biolog OmniLog incubator for 7 days (Dai et al., 2022). The Biolog OmniLog system was equipped with a charge-coupled device camera system and an incubator (Feng et al., 2023). A Heatmap Illustrator (HemI 1.0.3.3) was used to analyze the microbe carbon metabolism of the samples.

### 2.5 Statistical analysis

The mean value and standard error were used to express the measured results. One-way ANOVA or nonparametric test Kruskal–Wallis was used to compare the differences between multiple groups. *p* ≤ 0.05 was considered to be statistically significant.

## 3 Results

### 3.1 Quality of total bacterial and fungal sequence data

**Figure 2:**
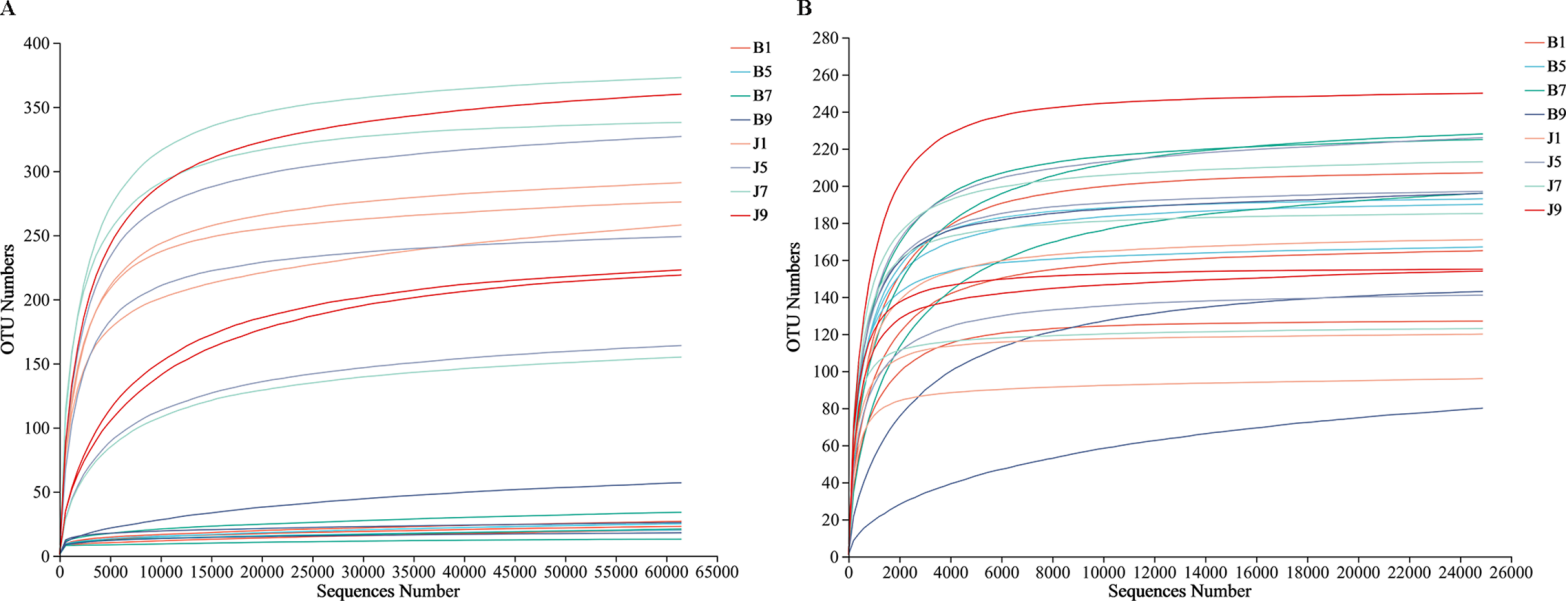
Rarefaction curves of bacterial **(A)** and fungal **(B)** OTUs across different tobacco leaf samples.

A total of 2,277,441 bacterial sequences were obtained through sequencing of 24 samples. These sequences were classified into 845 OTUs at a 97% similarity level. For fungal sequences, a total of 1,681,293 sequences were classified into 389 OTUs across the 24 samples. Rarefaction curves for each sample reached a plateau, indicating sufficient sequencing depth to capture most microorganisms present (**Figure 2**). All the bacterial and fungal sequences were deposited in the SRA database under accession numbers PRJNA1096149 and PRJNA1096455, respectively.

### 3.2 Distribution and diversity of bacterial and fungal operational taxonomic units

**Figure 3:**
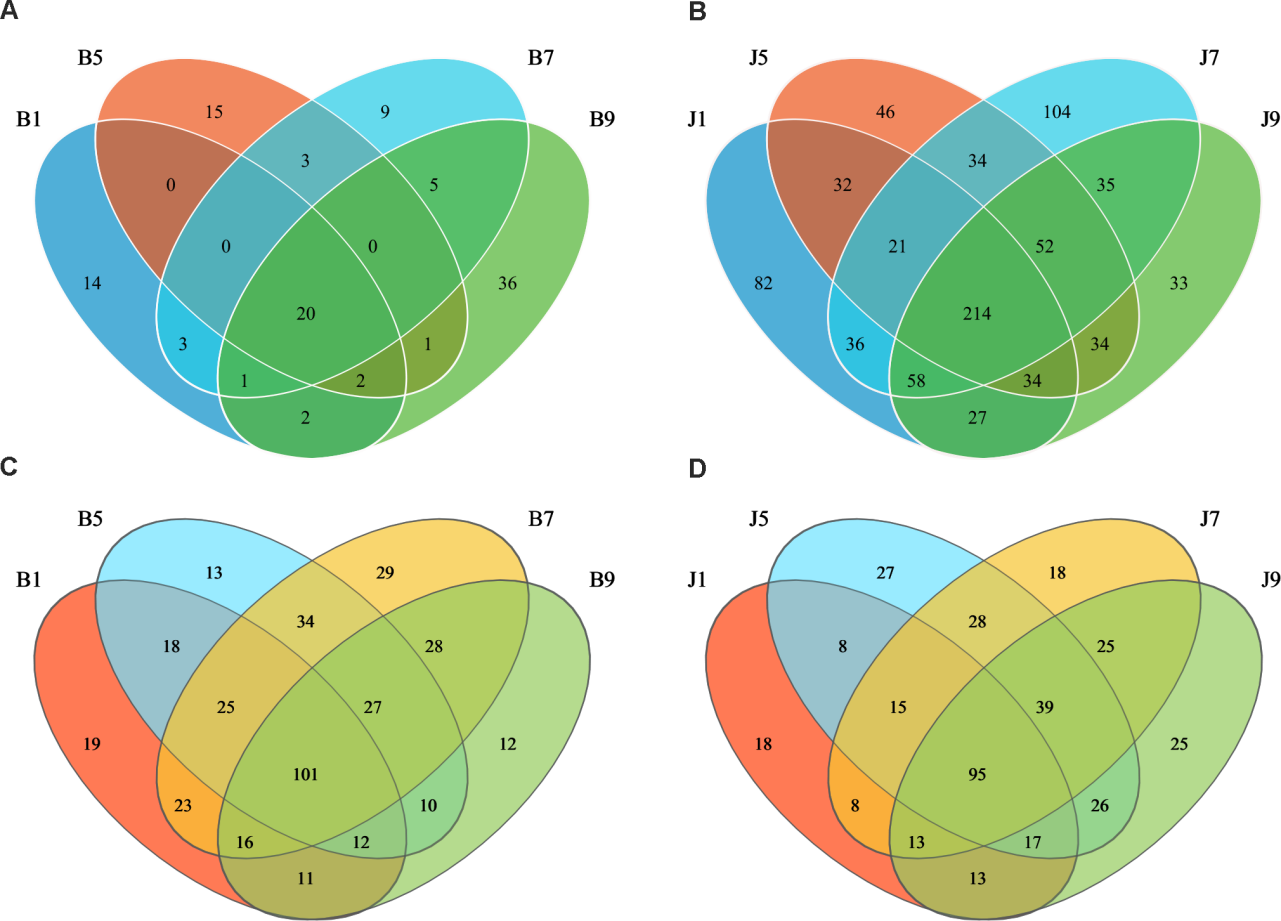
Venn diagram showing the number of bacterial and fungal OTUs observed in different groups at four different disease severity. Bacterial Venn diagram in diseased **(A)** and healthy **(B)** groups at four different disease severities. Fungal Venn diagram in diseased **(C)** and healthy **(D)** groups at four different disease severities. Numbers in the overlapping region indicated unique OTUs for two or three samples. Numbers in the non-overlapping regions indicated unique OTUs for the group.

Venn diagrams (**Figure 3**) show the distribution of the OTUs in the four disease severity levels. For bacterial community, a total of 845 OTUs were obtained, including 111 belonging to the diseased group and 842 to the healthy group (**Figures 3A, B**). The number of shared OTUs among the four diseased groups was 20; there were 14, 15, 9, and 36 unique OTUs found only in B1, B5, B7, and B9, respectively. Meanwhile, the number of shared OTUs among the four health groups was 214, and the groups J1, J5, J7, and J9 only had 82, 46, 104, and 33 unique OTUs, respectively. Notably, the number of bacterial OTUs in the diseased samples was lower than that in the healthy samples. We compared the bacterial Alpha diversity of the tobacco leaves using the diversity index (Shannon index) and the richness indices (Ace and Chao1 index) in **Table 2**. The higher the Shannon value, the greater the level of diversity. The higher the Ace and Chao1 value, the greater the level of richness. The Shannon index of diseased and healthy samples ranged from 1.31 ± 0.33 to 1.44 ± 0.17 and 2.11 ± 0.87 to 2.66 ± 1.44, respectively. The Ace index ranged from 31.34 ± 14.73 to 55.72 ± 37.05 and 332.30 ± 103.30 to 404.90 ± 170.80 for diseased and healthy samples, respectively, whereas the Chao1 index ranged from 26.42 ± 12.45 to 49.11 ± 36.39 for diseased and 334.30 ± 106.50 to 404.20 ± 166.70 for healthy samples. Correspondingly, the diversity indices Ace, Chao1, and Shannon of healthy samples were greater than those of diseased samples, and the diversity index decreased significantly with increased disease severity level from L1 to L5 and then increased with the increase in the degree of disease from L5 to L9.

**Table 2:** Alpha-diversity indices of bacterial and fungal communities based on high-throughput sequencing in different samples.

	Disease severity groups	Richness index		Diversity index	Coverage
		Ace	Chao1	Shannon	Coverage
Bacteria	B1	35.91 ± 1.24a	33.33 ± 1.86a	1.39 ± 0.28a	1.000
	B5	33.99 ± 7.97a	31.70 ± 7.43a	1.31 ± 0.33a	1.000
	B7	31.34 ± 14.73a	26.42 ± 12.45a	1.44 ± 0.17a	1.000
	B9	55.72 ± 37.05a	49.11 ± 36.39a	1.42 ± 0.23a	1.000
	J1	380.60 ± 34.04b	371.72 ± 21.74b	2.54 ± 0.28b	0.999
	J5	332.30 ± 103.30b	334.30 ± 106.50b	2.11 ± 0.87ab	0.999
	J7	404.90 ± 170.80b	404.20 ± 166.70b	2.66 ± 1.44ab	1.000
	J9	369.00 ± 111.40b	368.30 ± 108.00b	2.28 ± 0.77ab	0.999
Fungi	B1	125.50 ± 18.91a	127.00 ± 16.56a	2.62 ± 0.34a	1.000
	B5	133.10 ± 8.18a	132.90 ± 8.26a	3.54 ± 0.14a	1.000
	B7	171.60 ± 7.86a	175.60 ± 5.74a	3.11 ± 0.78a	1.000
	B9	121.30 ± 33.89a	121.40 ± 38.26a	2.21 ± 1.45a	0.999
	J1	94.78 ± 17.94a	94.78 ± 17.43a	3.02 ± 0.42a	1.000
	J5	146.00 ± 29.09a	147.30 ± 31.41a	3.55 ± 0.38a	1.000
	J7	132.30 ± 34.55a	133.80 ± 37.27a	3.77 ± 0.25a	1.000
	J9	138.20 ± 37.90a	138.30 ± 38.08a	3.41 ± 0.42a	1.000

Different letters in column direction represent having significant difference between groups (p <0.05).

In the fungal community, a total of 389 OTUs were obtained, with 378 OTUs in the diseased samples and 375 in the healthy group (**Figures 3C, D**). The four diseased and four healthy samples contained 101 and 95 shared OTUs, respectively. The unique OTU numbers in the four diseased groups were 19, 13, 29, and 12, respectively, while in the healthy group, the numbers were 18, 27, 18, and 25, respectively. The number of fungal OTUs in the diseased samples was higher than that in the healthy, but there was little difference. Similarly, the Shannon index of diseased and healthy samples ranged from 2.21 ± 1.45 to 3.54 ± 0.14 and 3.02 ± 0.42 to 3.77 ± 0.25, respectively (**Table 2**). The Ace index ranged from 121.30 ± 33.89 to 171.60 ± 7.86 and 94.78 ± 17.94 to 146.00 ± 29.09 for diseased and healthy samples, respectively, whereas the Chao1 index ranged from 121.40 ± 38.26 to 175.60 ± 5.74 for disease and 94.78 ± 17.43 to 147.30 ± 31.41 for healthy samples. The Ace and Chao1 indices were the highest at B7 and J5 in the disease and health groups, respectively. The Shannon index was the highest at B5 and J7 in the disease and health groups, respectively. Meanwhile, the Shannon index of healthy samples was greater than that of diseased samples, which indicated that the fungal diversity in the healthy group is higher than the diseased group.

### 3.3 Bacterial and fungal community composition

#### 3.3.1 Bacterial community composition

**Figure 4:**
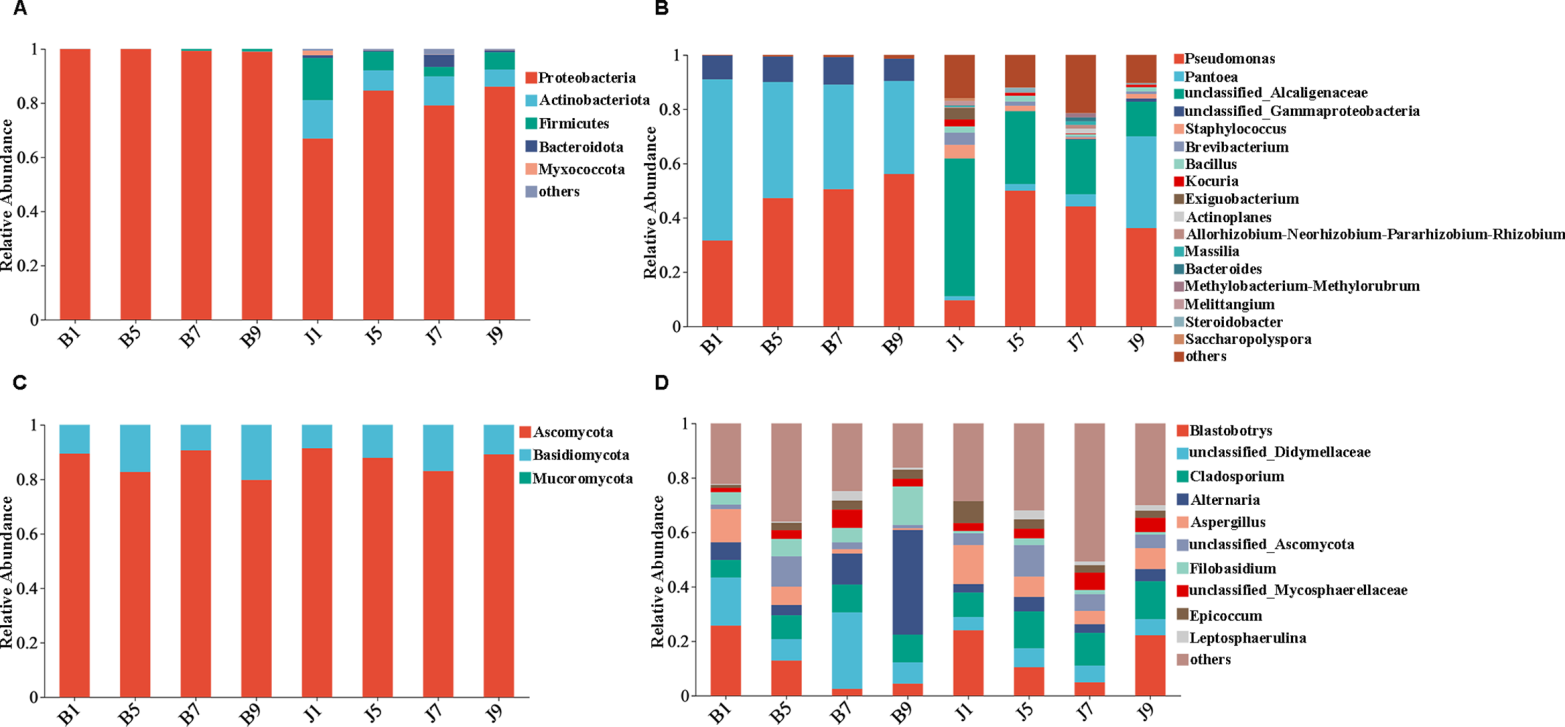
Microbial community composition of different groups at the phyla and genus levels. Bacterial community composition of different groups at the phyla **(A)** and genus **(B)** levels. Fungal community composition of different groups at the phyla **(C)** and genus **(D)** levels.

The 16S dataset showed that the phyllosphere bacteria of diseased and healthy samples contain six phyla of bacteria (**Table 3**): Proteobacteria, Actinobacteria, Firmicutes, Bacteroidetes, Myxococcota, and Chloroflexi (**Figure 4A**). Proteobacteria was the predominant phylum, with relative abundances in the diseased groups (B1, B5, B7, and B9) being 99.95%, 99.97%, 99.25%, and 98.97%, respectively, while in healthy groups (J1, J5, J7, and J9), the relative abundance were 66.89%, 84.56%, 79.12%, and 86.06%, respectively. The relative abundances of Actinobacteria in the diseased groups (B1, B5, B7, and B9) were 0.04%, 0.02%, 0.02%, and 0.08%, respectively; the relative abundances in healthy groups (J1, J5, J7, and J9) were 14.17%, 7.49%, 10.72%, and 6.31%, respectively. Notably, the relative abundance of Proteobacteria in diseased samples were higher than those in healthy samples. Conversely, the relative abundance of Actinobacteria and Firmicutes in diseased samples were less than in healthy samples.

**Table 3:** List of top 10 dominant taxa and their relative abundance in the fungal and bacterial communities of the group.

Community Structure		Relative abundance (%)							
Bacteria		B1	B5	B7	B9	J1	J5	J7	J9
Phylum	Proteobacteria	99.95 ± 0.07a	99.97 ± 0.01a	99.25 ± 0.78a	98.97 ± 1.54a	66.89 ± 10.72b	84.56 ± 8.07ab	79.12 ± 14.41ab	86.06 ± 13.65ab
	Actinobacteria	0.04 ± 0.06a	0.02 ± 0.01a	0.02 ± 0.02a	0.08 ± 0.07a	14.17 ± 4.25b	7.49 ± 5.00ab	10.72 ± 9.28ab	6.31 ± 7.47ab
	Firmicutes	0.01 ± 0.01a	0.01 ± 0.01a	0.71 ± 0.75a	0.94 ± 1.54a	15.57 ± 7.34b	6.92 ± 2.52abc	3.50 ± 1.54ac	6.40 ± 4.82abc
	Bacteroidetes	0.00 ± 0.00a	0.00 ± 0.00a	0.00 ± 0.00a	0.00 ± 0.00a	1.01 ± 0.16a	0.37 ± 0.09a	4.49 ± 5.35a	0.69 ± 0.74a
	Myxococcota	0.00 ± 0.00a	0.00 ± 0.00a	0.00 ± 0.00a	0.00 ± 0.00a	1.71 ± 2.64a	0.06 ± 0.08a	0.09 ± 0.08a	0.07 ± 0.11a
	Chloroflexi	0.00 ± 0.00a	0.00 ± 0.00a	0.00 ± 0.00a	0.00 ± 0.00a	0.31 ± 0.34a	0.21 ± 0.18a	0.68 ± 0.59a	0.28 ± 0.46a
Genus	*Pseudomonas*	31.59 ± 26.57a	47.24 ± 30.78a	50.53 ± 20.68a	56.10 ± 5.29a	9.58 ± 5.96a	49.98 ± 18.98a	44.15 ± 28.30a	36.18 ± 20.98a
	*Pantoea*	59.39 ± 30.33a	42.74 ± 30.73ab	38.51 ± 20.71ab	34.28 ± 0.19ab	1.50 ± 0.96b	2.46 ± 1.97b	4.51 ± 3.94b	33.80 ± 7.45ab
	*Unclassified Alcaligenaceae*	0.02 ± 0.02a	0.04 ± 0.03a	0.01 ± 0.02a	0.01 ± 0.01a	50.77 ± 11.45b	26.83 ± 7.03c	20.15 ± 9.41c	12.78 ± 4.46ac
	*Unclassified Gammaproteobacteria*	8.74 ± 4.20a	9.40 ± 2.25a	10.12 ± 1.01a	8.21 ± 4.30a	0.01 ± 0.01b	0.05 ± 0.01b	0.28 ± 0.12b	1.16 ± 0.92b
	*Staphylococcus*	0.00 ± 0.00a	0.00 ± 0.00a	0.00 ± 0.00a	0.00 ± 0.00a	5.00 ± 3.49b	1.95 ± 0.24ab	0.48 ± 0.18a	1.70 ± 1.75ab
	*Brevibacterium*	0.00 ± 0.00a	0.00 ± 0.00a	0.00 ± 0.00a	0.00 ± 0.00a	4.46 ± 1.65b	1.55 ± 1.55ac	0.32 ± 0.31ac	0.91 ± 0.75ac
	*Bacillus*	0.00 ± 0.00a	0.01 ± 0.01a	0.00 ± 0.00a	0.01 ± 0.02a	2.31 ± 1.03a	2.14 ± 2.26a	0.89 ± 0.31a	1.59 ± 0.53a
	*Kocuria*	0.00 ± 0.00a	0.00 ± 0.00a	0.00 ± 0.00a	0.00 ± 0.00a	2.58 ± 1.70b	1.06 ± 0.39ab	0.28 ± 0.07a	0.92 ± 1.03ab
	*Exiguobacterium*	0.00 ± 0.00a	0.00 ± 0.00a	0.00 ± 0.00a	0.00 ± 0.00a	4.26 ± 7.32a	0.01 ± 0.01a	0.02 ± 0.02a	0.03 ± 0.05a
	*Actinoplanes*	0.00 ± 0.00	0.00 ± 0.00	0.00 ± 0.00	0.00 ± 0.00	0.03 ± 0.02	0.09 ± 0.12	1.79 ± 3.07	0.06 ± 0.09
Fungi		B1	B5	B7	B9	J1	J5	J7	J9
Phylum	Ascomycota	89.43 ± 7.14a	82.68 ± 9.21a	90.60 ± 3.61a	79.75 ± 12.56a	91.37 ± 0.45a	87.87 ± 4.54a	83.02 ± 3.78a	89.12 ± 2.94a
	Basidiomycota	10.56 ± 7.13a	17.32 ± 9.20a	9.40 ± 3.61a	20.25 ± 12.56a	8.62 ± 0.45a	12.12 ± 4.54a	16.98 ± 3.78a	10.88 ± 2.94a
	Mucoromycota	0.01 ± 0.01a	0.00 ± 0.01a	0.00 ± 0.00a	0.00 ± 0.00a	0.01 ± 0.01a	0.00 ± 0.00a	0.00 ± 0.00a	0.00 ± 0.00a
Genus	*Blastobotrys*	25.70 ± 19.68a	12.87 ± 5.19a	2.48 ± 1.67a	4.41 ± 4.36a	24.00 ± 18.00a	10.43 ± 6.41a	4.88 ± 2.74a	22.15 ± 10.54a
	*unclassified_Didymellaceae*	17.69 ± 23.93a	7.90 ± 4.78a	28.09 ± 21.62a	7.79 ± 9.32a	4.88 ± 1.34a	6.95 ± 2.69a	6.15 ± 1.52a	5.94 ± 2.28a
	*Cladosporium*	6.45 ± 1.14a	8.75 ± 3.21a	10.22 ± 2.36a	10.20 ± 4.85a	9.01 ± 7.45a	13.55 ± 2.86a	12.04 ± 5.91a	13.93 ± 2.54a
	*Alternaria*	6.47 ± 8.31a	3.76 ± 0.71a	11.41 ± 13.90a	38.43 ± 35.49a	3.08 ± 0.93a	5.33 ± 3.18a	3.17 ± 1.73a	4.48 ± 1.68a
	*Aspergillus*	12.19 ± 9.62a	6.74 ± 4.40a	1.59 ± 0.65a	0.65 ± 0.64a	14.34 ± 9.58a	7.46 ± 4.36a	4.87 ± 3.99a	7.66 ± 3.35a
	*unclassified_Ascomycota*	1.66 ± 0.91a	11.15 ± 11.91a	2.48 ± 0.50a	1.20 ± 1.03a	4.31 ± 1.00a	11.57 ± 6.52a	6.14 ± 2.96a	4.97 ± 3.50a
	*Filobasidium*	4.56 ± 3.20a	6.40 ± 7.23a	5.32 ± 2.80a	14.15 ± 15.88a	0.84 ± 0.57a	2.46 ± 3.07a	1.55 ± 2.35a	0.94 ± 1.14a
	*unclassified_Mycosphaerellaceae*	1.59 ± 0.70a	3.21 ± 2.63a	6.75 ± 3.30a	2.82 ± 4.40a	2.92 ± 2.72a	3.55 ± 0.97a	6.37 ± 3.20a	5.25 ± 1.42a
	*Epicoccum*	1.09 ± 0.74a	2.70 ± 0.39a	3.30 ± 1.01a	3.36 ± 2.59a	8.05 ± 6.97a	3.44 ± 1.15a	2.76 ± 1.00a	2.64 ± 1.42a
	*Leptosphaerulina*	0.30 ± 0.41a	0.43 ± 0.31a	3.48 ± 5.22a	0.73 ± 1.11a	0.00 ± 0.00a	3.30 ± 5.12a	1.36 ± 2.12a	2.00 ± 3.00a

Different letters in column direction represent having significant difference between groups (p <0.05).

At the genus level, the top 10 genera included *Pseudomonas*, *Pantoea*, unclassified_Alcaligenaceae, unclassified_Gammaproteobaceria, *Staphylococcus*, *Brevibacterium*, *Bacillus*, *Kocuria*, *Exiguobacteri*, and *Actinoplanes* (**Figure 4B**). The abundance of *Pseudomonas*, *Pantoea*, and unclassified_Gammaproteobaceria in diseased samples was higher than that in healthy samples, whereas the abundance of unclassified_ Alcaligenaceae in diseased samples was less than that in healthy samples. The abundance of *Pseudomonas* increased with the increase in disease severity level and peaked at L9 sample. On the contrary, the abundance of *Pantoea* decreased with the increase in disease severity level (**Table 3**). In addition, with the increase in disease severity level, the relative abundance of unclassified_Alcaligenaceae decreased. The result revealed that the bacterial community composition was different for four disease severity levels.

#### 3.3.2 Fungal community composition

The ITS dataset showed that the phyllosphere fungi of diseased and healthy samples contain three phyla of fungi: Ascomycota, Basidiomycota, and Mucoromycota (**Figure 4C**). The predominant fungal phylum was Ascomycota, with relative abundances in the diseased groups (B1, B5, B7, and B9) were 89.43%, 82.68%, 90.60%, and 79.75%, respectively, while in healthy groups (J1, J5, J7, and J9), the relative abundances were 91.37%, 87.87%, 83.02%, and 89.12%, respectively. The relative abundances of Basidiomycota in the diseased groups (B1, B5, B7, and B9) were 10.56%, 17.32%, 9.40%, and 20.25%, respectively; the relative abundances in healthy groups (J1, J5, J7, and J9) were 8.62%, 12.12%, 16.98%, and 10.88%, respectively (**Table 3**). The result indicates that the relative abundance of Ascomycota in diseased samples was lower than that in healthy samples, and the relative abundance of Basidiomycota in diseased samples was higher than that in healthy samples, except for L7 samples.

At the genus level, the top 10 genera included *Blastobotrys*, unclassified_Didymellaceae, *Cladosporium*, *Alternaria*, *Aspergillus*, unclassified_Ascomycota, *Filobasidium*, unclassified_Mycosphaerellaceae, *Epicoccum*, and *Leptosphaerulina* (**Figure 4D**). Similarly, the relative abundance was associated with the four disease severity levels samples, but the difference is not significant. The abundance of unclassified_Didymellaceae, *Alternaria*, and *Filobasidium* in diseased samples was higher than that in healthy samples, whereas the abundance of *Cladosporium* and *Aspergillus* in diseased samples was less than that in healthy samples (**Table 3**). The abundance of *Aspergillus* decreased with the increase in disease severity level in diseased samples.

### 3.4 Spatial distribution of bacterial and fungal communities

**Figure 5:**
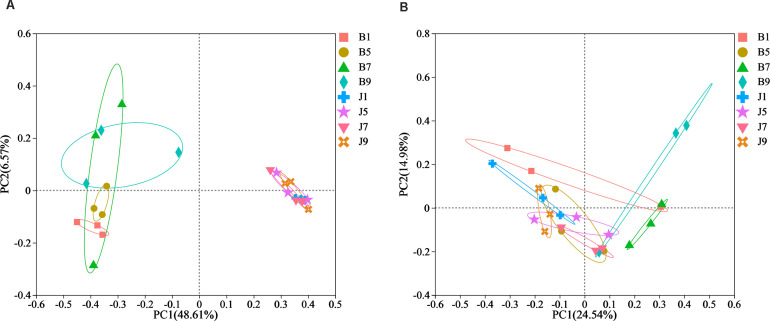
Principal Co-ordinate Analysis (PCoA) of the bacterial **(A)** and fungal **(B)** communities in the different group sample.

PCoA plots were used to reveal the spatial distribution of fungal and bacterial communities (**Figure 5**). In bacterial community, the result showed that diseased groups and healthy groups were obviously separate (**Figure 5A**). All diseased samples (B1, B5, B7, and B9) from four disease severity levels were irregularly separated from each other. On the contrary, healthy groups (J1, J5, J7, and J9) with four disease severity levels were clustered together. In the fungal community, healthy groups (J1, J5, J7, and J9) with four disease severity levels were clustered together, and diseased samples (B1, B5, B7, and B9) from four disease severity levels were irregularly separated from each other (**Figure 5B**). Notably, B1 and B5 of the diseased group were clustered together with healthy groups; B7 and B9 were separated from the healthy group. The result indicated that the spatial distributions of bacterial and fungal community composition were significantly different between disease and healthy samples. There were fewer differences in bacterial and fungal community composition of healthy samples compared to diseased samples.

### 3.5 Significant differences in microbial communities

**Figure 6:**
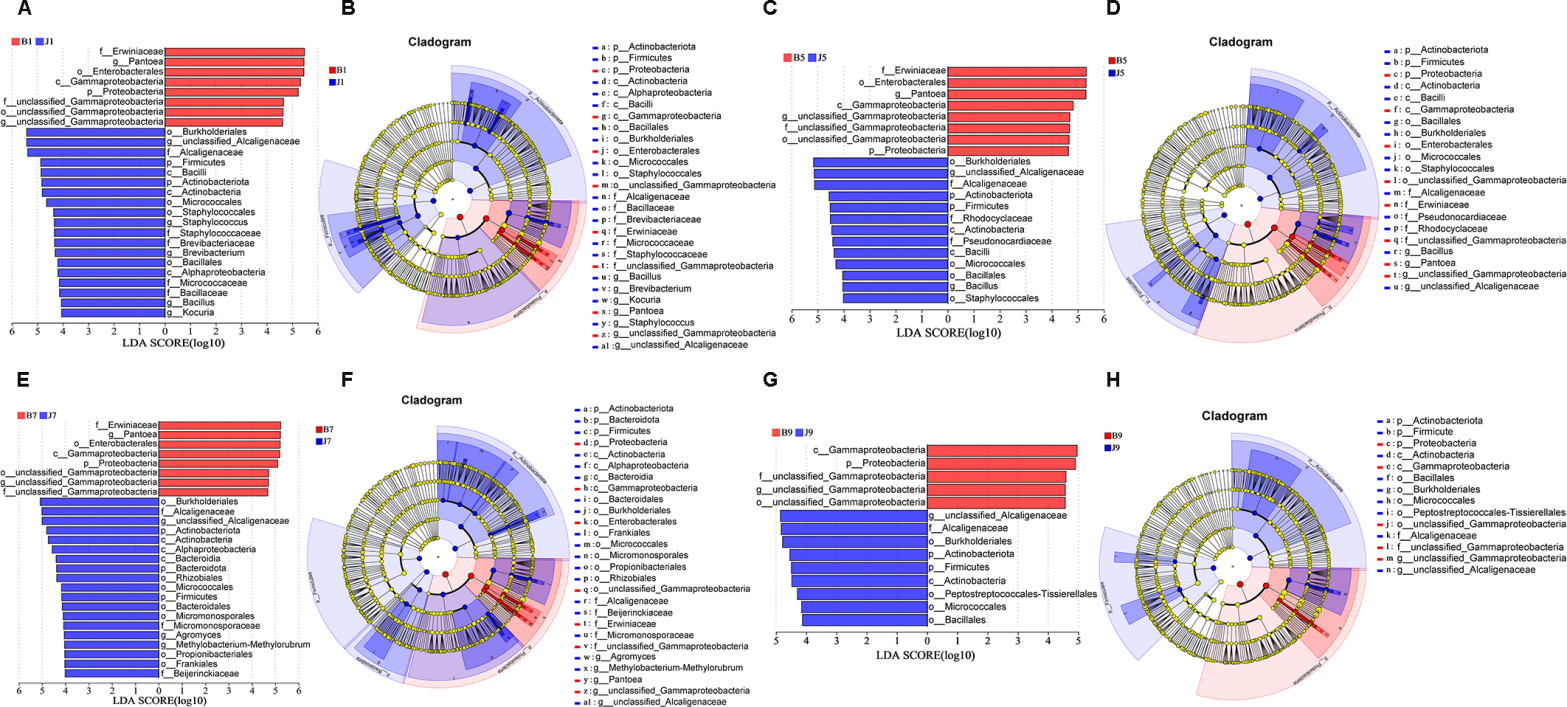
LEfSe analysis of bacteria abundance between diseased and healthy samples of different disease severities. **(A)**, **(C)**, **(E)**, **(G)** are LDA score identified the size of differentiation between diseased and healthy groups of 1, 5, 7, 9 disease severity, respectively, with a threshold value of 4. **(B)**, **(D)**, **(F)**, **(H)** are the cladogram of microbial communities of 1, 5, 7, 9 disease severity, respectively.

**Figure 7:**
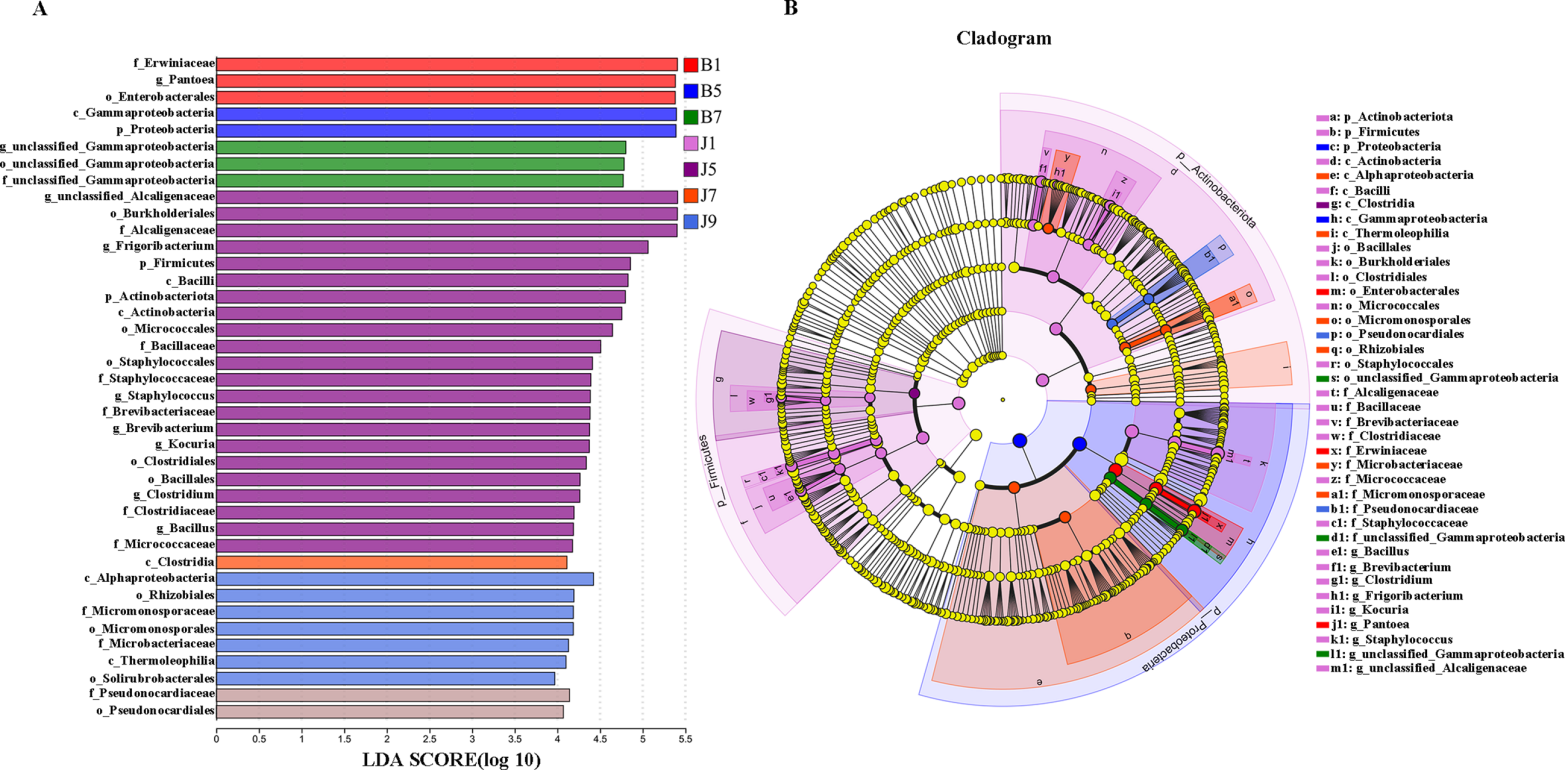
LEfSe analysis of bacteria abundance in all samples, LDA score **(A)** and cladogram of microbial communities **(B)**.

LEfSe analysis was used to detect the difference in fungal and bacterial communities between the diseased and healthy samples (**Figure 6**). For bacterial communities, 89 bacteria clades showed statistically significant differences when the LDA threshold was 4. In L1 leaf samples, 19 and 8 clades showed abundance advantage in healthy and diseased samples, respectively (**Figures 6A, B**). In L5 leaf samples, 13 and 8 clades present an abundance advantage in healthy and diseased samples, respectively (**Figures 6C, D**). In L7 leaf samples, 19 clades showed abundance advantage in healthy samples, while eight clades showed abundance advantage in diseased samples (**Figures 6E, F**). In L9 leaf samples, nine and five clades present abundance advantage in healthy and diseased samples, respectively (**Figures 6G, H**). At the genus level, *Pantoea* and unclassified_Gammaproteobacteria were enriched in B1, B5, and B7 samples, while the B9 sample only enriched unclassified_Gammaproteobacteria. Unclassified_Alcaligenaceae were enriched in J1, J5, J7, and J9 samples. Meanwhile, J1 healthy samples also were enriched with *Staphylococcus*, *Brevibacterium*, *Bacillus*, and *Kocuria*; J5 healthy samples also were enriched with *Bacillus*; and J7 healthy samples also were enriched with *Methylobacterium–Methylorubrum*. At the same time, significant differences were found between the different treatment groups using PERMANOVA test. The healthy groups had more characteristic species compared to the diseased groups. However, the B9 did not have characteristic species (**Figure 7**). Based on the results of LEfSe analysis, we found differences between healthy and diseased groups, and differences between samples of different disease severity levels. In addition, it was observed that the diversity of the enriched bacterial species was lower as the disease levels L1–L9 increased, indicating that the disease level has a strong influence on bacterial diversity. In contrast, there were no significant abundance differences for fungal communities between the diseased and healthy microbial communities of different disease severity levels.

### 3.6 Microbial community functional characteristics

**Figure 8:**
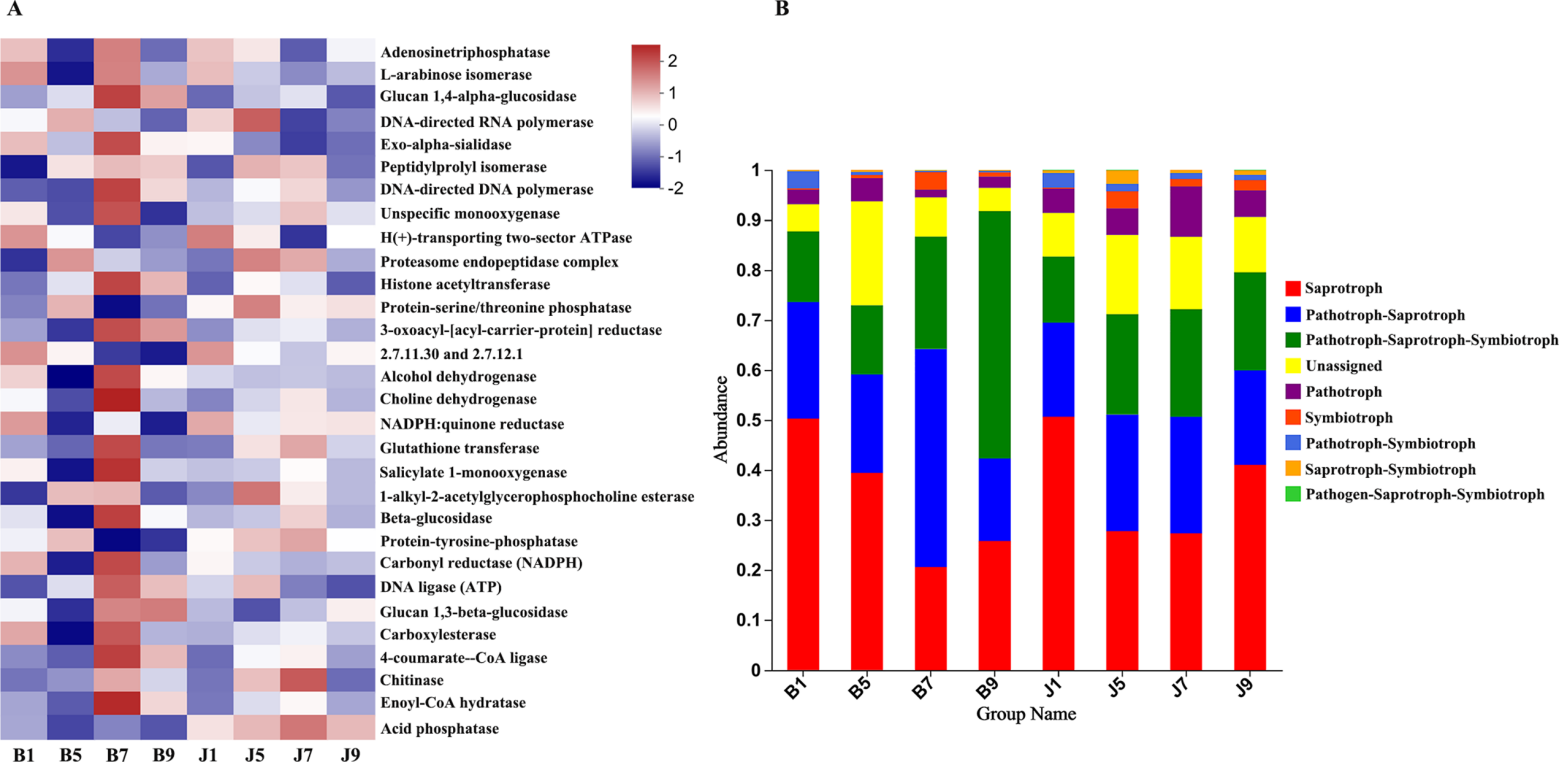
PICRUSt analyses of the enzyme functional of fungal in tobacco leaf samples **(A)**. Relative abundance of fungal functional groups (modes) based on OTU annotation table with disturbance frequency level **(B)**.

PICRUSt and FUNGuild were used to predict the trophic modes of fungi in different disease severity levels, respectively (**Figure 8**). The PICRUSt analyses result showed that the top 6 enzymes were Adenosinetriphosphatase, L-arabinose isomerase, Glucan 1,4-alpha-glucosidase, DNA-directed RNA polymerase, Exo-alpha-sialidase, and unspecific monooxygenase. Meanwhile, most of the enzymes did not differ significantly between treatment groups. At the FUNGuild result, saprotroph, pathotroph–saprotroph, and pathotroph–saprotroph–symbiotroph were the dominant trophic modes in all samples. The abundance of saprotroph was higher in healthy groups than that in diseased groups and decreased with the increase in disease severity level. In addition, pathotroph–saprotroph–symbiotroph had a higher abundance in diseased groups compared to healthy groups, which increased with increasing disease severity level.

**Figure 9:**
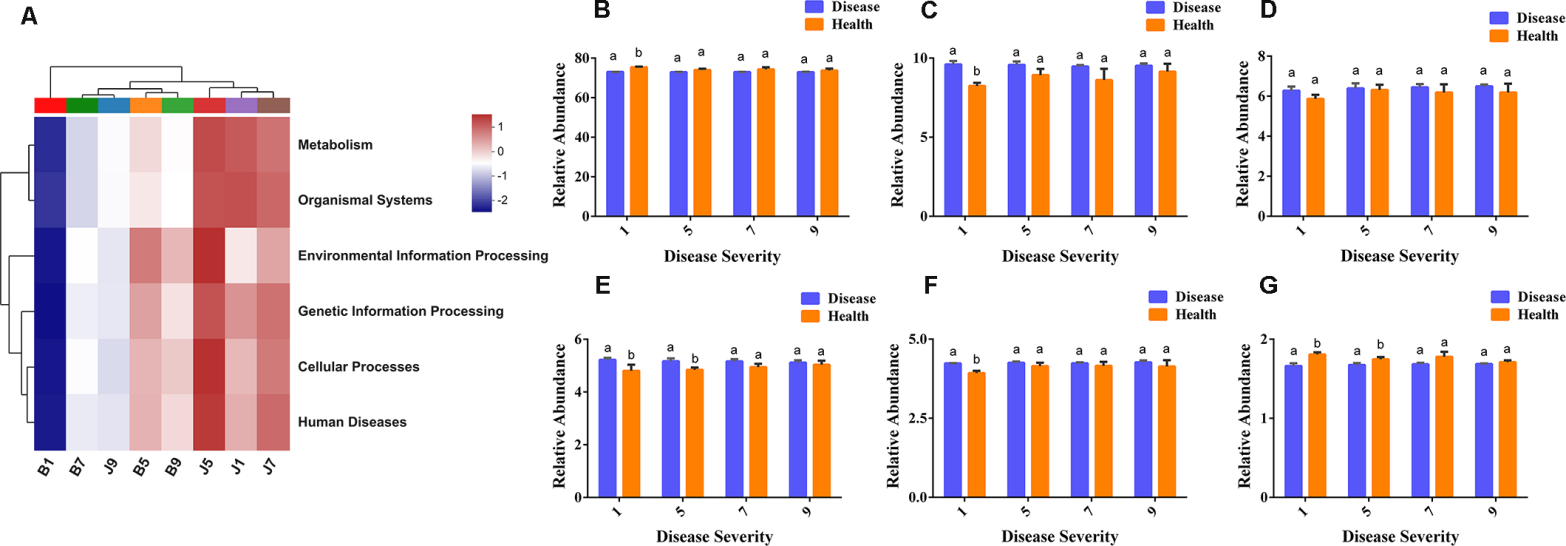
PICRUSt analyses of the KEGG level 1 functional categories of bacteria in tobacco leaf samples. **(A)** Dendrogram of bacterial functional categories, **(B)** Metabolism, **(C)** Environmental Information Processing, **(D)** Cellular Processes, **(E)** Genetic Information Processing, **(F)** Human Diseases, **(G)** Organismal Systems.

PICRUSt2 was used to predict the bacterial community function. In this study, the most abundant pathway was metabolic (73.67%), followed by environmental information processing (9.14%), cellular processes (6.27%), genetic information processing (5.04%), and human disease (4.17%), with the lowest being the organic system (1.72%). B1 samples were the lowest for all functions, and J9 samples clustered with the diseased samples, which indicates that L9 samples were the most severely diseased, and the bacterial functions in their healthy samples were similar to those of the diseased samples (**Figure 9A**). There were significant differences between diseased and healthy samples at L1, except for cellular processes. However, most bacterial functions seem to be independent of the disease severity level (**Figures 9B–G**).

### 3.7 Microbial co-occurrence network analysis

**Figure 10:**
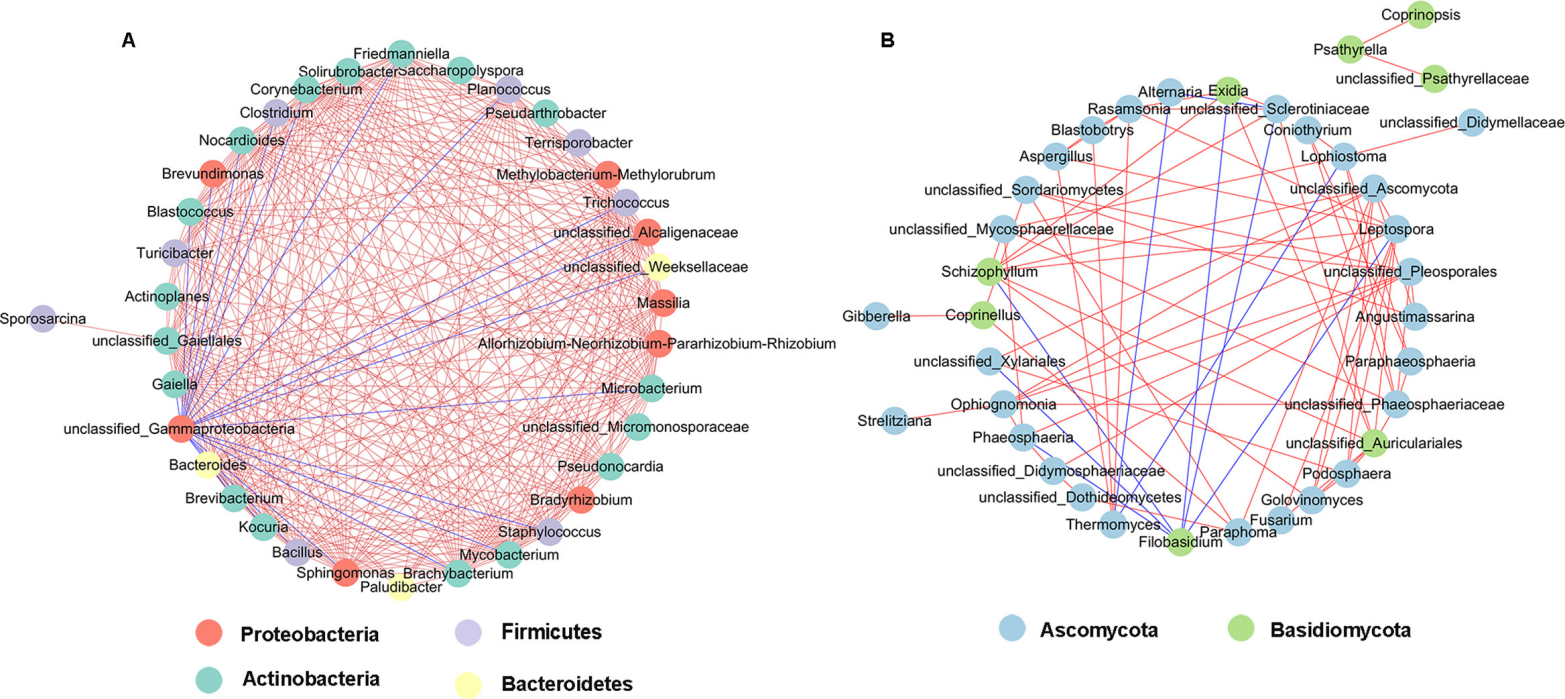
Network analysis of bacterial **(A)** and fungal **(B)** taxa showing significant positive or negative co-occurrences based on Spearman’s rank correlations. Red lines represent positive correlations and blue lines represent negative correlations.

Network analysis of co-occurrence patterns among the most abundant 50 bacterial and fungal genera presents that most of the bacterial genera showed positive correlation with each other, and unclassified_Gammaproteobacteria showed negative correlation with 19 bacterial genera such as unclassified_Alcaligenaceae, *Kocuria*, *Bacillus*, and *Brevibacterium* (**Figure 10A**). For fungal communities, most of the fungal genera were also positively correlated; *Filobasidium* was negatively correlated with six genera including *Phaeosphaeria*, *Leptospora*, and *Exidia*, and *Alternaria* was negatively correlated with *Thermomyces* and Sclerotiniaceae (**Figure 10B**).

### 3.8 Microbial community metabolic profiles

**Figure 11:**
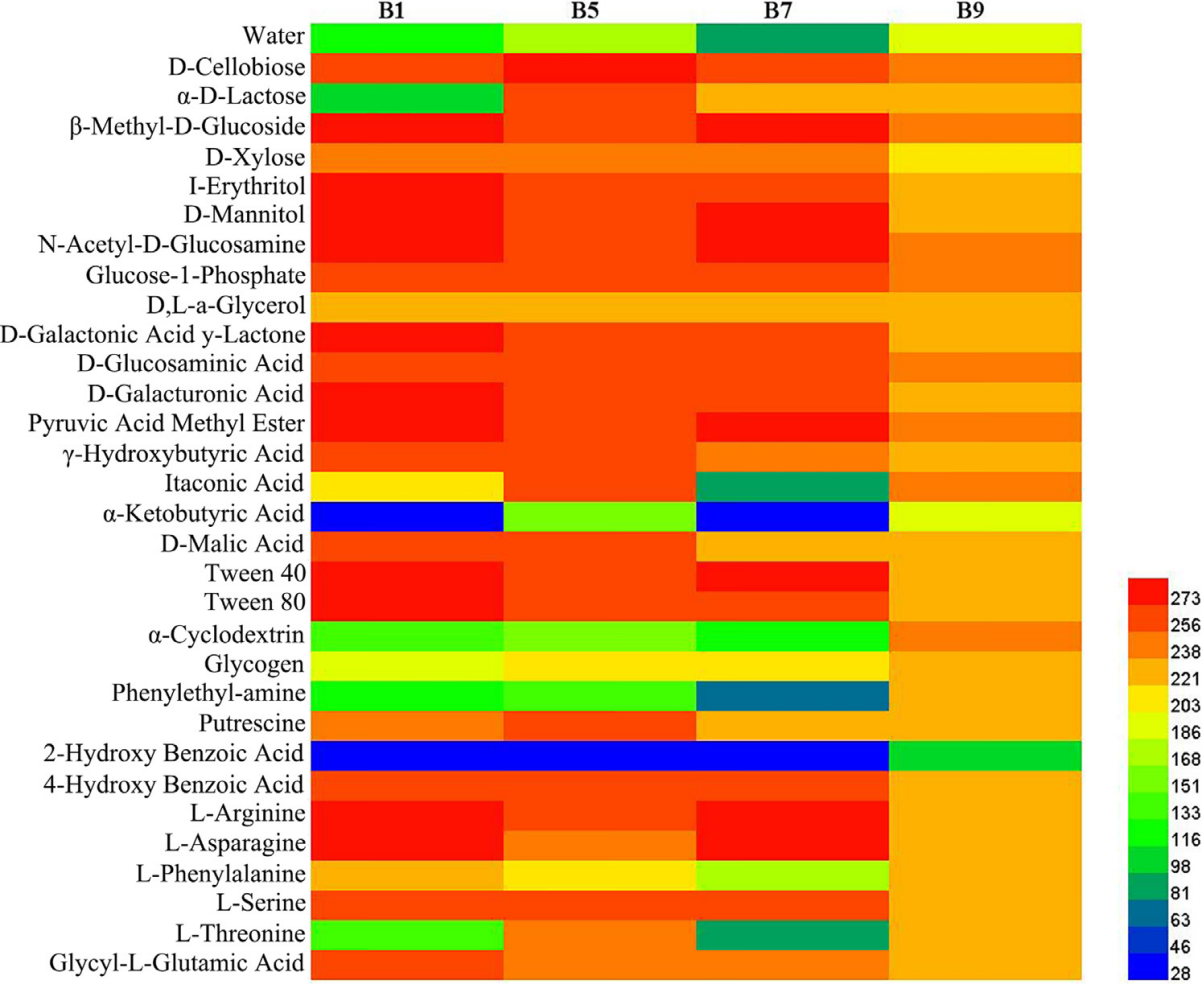
Heatmap of carbon sources in BIOLOG ECO-Plate of microbial communities from different samples.

Phyllosphere microbial metabolic function analysis was performed using Biolog Eco metabolic 96-well plates (**Figure 11**). The metabolic capacity of carbon sources of different disease level samples decreased with increasing disease level, with B9 samples having the lowest metabolic capacity. The samples with different disease levels had lower capacity to utilize 2-hydroxybenzoic acid, α-cyclodextrin, α-butyronic acid, and phenylethyl-amine, but the B9 sample had a greater capacity to utilize these carbon sources than the other disease level samples.

## 4 Discussion

Disease severity level is an important factor to affect the structure of microbial communities in tobacco leaves. Understanding of the microbial characteristics of tobacco leaves with different disease severity levels is of great value to disease control. In this study, high-throughput sequencing and Biolog Eco were used to investigate the impact of disease severity level of tobacco wildfire disease on microbial communities and carbon source metabolizing capacity. It was shown that healthy plant leaves harbored greater microbial abundances and diversity compared to diseased leaves, with a highly diverse microbial diversity that tends to be conducive to resisting pathogen infection (De Mandal and Jeon, 2023). Similar results have been reported in this study, the Shannon, Ace, and Chao1 indices of bacterial community were higher in healthy groups than the diseased groups, and the Shannon index of the fungal community were higher in healthy groups than that diseased groups. The results suggested that the healthy phyllosphere has greater microbial diversity to resist disease expansion. Moreover, the Shannon index of bacteria decreased and then increased with increasing disease severity level, which was consistent with the conclusion of bacterial community diversity at different disease levels of powdery mildew in pumpkin reported by Luo et al. (2017). Conversely, Zhang et al. (2019) reported that the fungal diversity index showed increasing and then decreasing trend with the aggravation of the disease, which was consistent with the conclusion in this study about the diversity index of fungi. In general, the results suggested that there may be an interactive relationship between the bacteria and fungi in the leaf of tobacco wildfire disease.

The composition of the microbial community could be affected by the colonization of pathogens. In bacterial communities, Proteobacteria was the dominant phylum in both diseased and healthy samples at four disease severity levels. The abundance of Proteobacteria was higher in diseased samples than in the healthy samples (Guo et al., 2023), whereas the abundance of Actinobacteria and Firmicutes was much less in diseased than in healthy samples. The results of a previous study showed that Actinobacteria activates biosynthesis of salicylic acid to induce defense responses in plant leaves (Vergnes et al., 2020). Proteobacteria and Firmicutes are the common bacterial phyla in the phyllosphere; they always show opposite trends, which is thought to be due to the fact that Firmicutes were inhibited by Proteobacteria (Chen et al., 2020b). This may explain why the abundance of Firmicutes in diseased samples was lower than in healthy samples in tobacco wildfire disease. The dominant bacterial genus of both diseased and healthy leaves was *Pseudomonas*, which was the genus to which the pathogen of tobacco wildfire disease belongs. On the other hand, some strains of *Pseudomonas* are thought to produce a wide range of antibiotics that are effective in suppressing agricultural diseases.

The result of this study showed that a high abundance of *Pseudomonas* may be caused by pathogenic colonization or by antimicrobial active strains recruited in the phyllosphere. In addition, the relative abundance of *Pseudomonas* positively correlated with the disease severity level in diseased samples, which is similar to the results reported by Sun et al. (2022) who pointed out that the abundance of *Thanatephorous* (pathogen of tobacco target spot disease) increased with the increase in disease severity level. The abundance of unclassified_Alcaligenaceae was higher in healthy samples compared to diseased samples, which decreased with increasing disease severity level. *Alcaligens* has been heavily reported as a biocontrol agent against tobacco diseases. The result of this study suggests that there are several potentially beneficial genera in healthy samples, and whether plant health can be maintained by maintaining the abundance of *Alcaligens* is very much worthy of further investigation.

In fungal communities, the dominant phyla were Ascomycota and Basidiomycota in both diseased and healthy leaves, which is similar to Liu et al. (2022). The taxa unclassified_Didymellaceae, *Alternaria*, and *Filobasidium* were more enriched in diseased samples, whereas the abundance of *Aspergillus* was higher in healthy samples. Si et al. (2023) reported that *Aspergillus* was higher in healthy samples compared to diseased samples, which is consistent with our study. Among them, the relative abundance of *Aspergillus* negatively correlated with the disease severity level in diseased samples. Huang et al. (2021) found that the relative abundance of *Alternaria* increased with a disease severity level and deduced that *Alternaria* could benefit from the disease pressure caused by *Didymella*. The results of this study were consistent with them, and it is possible that the occurrence of wildfire disease is beneficial to *Alternaria* growth, but it remains to be investigated whether this genus accelerates or reduces wildfire disease occurrence. Based on the functional predictions of FUNGuild, most fungal communities were identified as saprotroph and pathotroph–saprotroph. Pathotroph–saprotroph and pathotroph–saprotroph–symbiotroph had a higher abundance in diseased samples. The data signify the presence of a large number of fungi in tobacco leaf, having pathogenic potential.

LEfSe analysis showed that the *Pantoea* and unclassified_Gammaproteobacteria were enriched in the L1, L5, and L7 diseased groups of different disease severity levels, but the L9 diseased groups were only enriched with unclassified_Gammaproteobacteria. The healthy groups were all enriched unclassified_Alcaligenaceae; moreover, the L1 healthy sample was enriched for the genera *Brevibacterium*, *Bacillus*, and *Kocuria*, the L5 healthy sample was also enriched for *Bacillus*, the L7 healthy sample *Methylobacterium–Methylorubrum* was enriched, while the L9 healthy sample did not have any other characterized genera. The results present that the diversity of enriched microbial communities were decreased with increasing disease severity level. In previous studies, *Bacillus*, *Methylobacterium–Methylorubrum*, *Brevibacterium*, and unclassified_Alcaligenaceae were considered to be biocontrol agents, beneficial to plant growth and to protect plants from pathogens (Madhaiyan et al., 2007; Son et al., 2014). *Gammaproteobacteria* are common bacteria in plants; in this study unclassified_Gammaproteobacteria were found to be in higher abundance in diseased samples compared to healthy samples, which is consistent with previous studies (Wang et al., 2022). Furthermore, the results of correlation network analysis also showed that unclassified_Gammaproteobacteria showed negative correlation with unclassified_Alcaligenaceae, *Kocuria*, *Bacillus*, and *Brevibacterium* in healthy groups. *Gammaproteobacteria* are eutrophic bacteria (Huang et al., 2017); we speculate that their lower abundance in healthy samples may be due to the higher microbial abundance and diversity in healthy samples, which compete with *Gammaproteobacteria* for nutrients.

The result of substrate utilization profiling displayed that the metabolizing ability of all samples to most carbon sources decreased with increasing disease severity level, but the metabolizing ability to α-butyronic acid, α-cyclodextrin, glycogen, phenylethylamine, and 2-hydroxybenzoic acid increased with increasing disease severity level, which was similar to the change in *Pseudomonas* abundance, but the relationship between these carbon sources and *Pseudomonas* needs to be further investigated. Meanwhile, we found that all samples had the lowest utilization of 2-2hydroxybenzoic acid, which has been reported to play an important role in the regulation of plant growth and development and defense response against pathogenic bacteria (Miura and Tada, 2014), which may explain its low utilization.

## 5 Conclusion

In this study, we found that the bacterial and fungal abundance and diversity from diseased samples was less than in healthy samples in the leaves affected by tobacco wildfire disease. Moreover, the abundance of bacterial and fungal community composition and diversity was affected by disease severity level. The relative abundance of *Pseudomonas* positively correlated with the disease severity level in diseased samples. The bacterial diversity and relative abundance decrease at the beginning and then increase with the tobacco wildfire disease severity level increases. Nevertheless, the fungal diversity and relative abundance increase and then decrease with increasing disease severity level. Finally, the metabolizing ability of all samples to most carbon sources decreased with increasing disease severity level. The most significant differences were found in β-methyl-D-glucoside, D-mannitol, N-acetyl-D-glucosamine, D-galacturonic acid, pyruvic acid methyl ester, and Tween 40. The obtained data from this study helps to infer that the composition and diversity of phyllosphere microbiome in tobacco wildfire disease. However, further study is needed to verify the interaction of phyllosphere microbiome. Hence, it is essential to direct attention toward these characteristic microorganisms in further studies.

## Data Availability

The datasets presented in this study can be found in online repositories. The names of the repository/repositories and accession number(s) can be found below: https://www.ncbi.nlm.nih.gov/, PRJNA1096149 and PRJNA1096455.
